# From Entropy Generation to Exergy Efficiency at Varying Reference Environment Temperature: Case Study of an Air Handling Unit

**DOI:** 10.3390/e21040361

**Published:** 2019-04-03

**Authors:** Giedrė Streckienė, Vytautas Martinaitis, Juozas Bielskus

**Affiliations:** Department of Building Energetics, Vilnius Gediminas Technical University, Sauletekio ave. 11, LT-10223 Vilnius, Lithuania

**Keywords:** heating, ventilation, and air conditioning (HVAC), air handling unit, energy efficiency, exergy efficiency, produced entropy, variable reference temperature, coenthalpy

## Abstract

The continuous energy transformation processes in heating, ventilation, and air conditioning systems of buildings are responsible for 36% of global final energy consumption. Tighter thermal insulation requirements for buildings have significantly reduced heat transfer losses. Unfortunately, this has little effect on energy demand for ventilation. On the basis of the First and the Second Law of Thermodynamics, the concepts of entropy and exergy are applied to the analysis of ventilation air handling unit (AHU) with a heat pump, in this paper. This study aims to develop a consistent approach for this purpose, taking into account the variations of reference temperature and temperatures of working fluids. An analytical investigation on entropy generation and exergy analysis are used, when exergy is determined by calculating coenthalpies and evaluating exergy flows and their directions. The results show that each component of the AHU has its individual character of generated entropy, destroyed exergy, and exergy efficiency variation. However, the evaporator of the heat pump and fans have unabated quantities of exergy destruction. The exergy efficiency of AHU decreases from 45–55% to 12–15% when outdoor air temperature is within the range of −30 to +10 °C, respectively. This helps to determine the conditions and components of improving the exergy efficiency of the AHU at variable real-world local climate conditions. The presented methodological approach could be used in the dynamic modelling software and contribute to a wider application of the Second Law of Thermodynamics in practice.

## 1. Introduction

Buildings are one of the largest global energy consumers, using about 36% of final energy and responsible for nearly 40% of energy-related CO_2_ emissions [1]. Continuous energy transformations take place in heating, ventilation, and air conditioning (HVAC) systems and devices thereof. Tighter thermal insulation requirements for buildings have significantly reduced heat transfer losses through the building envelope. However, in order to ensure adequate air quality in buildings, ventilation systems are becoming more and more important, leading to an increased need to improve and more thoroughly analyze such systems.

When analyzing HVAC systems, we can distinguish two large general groups of energy transformers: The first—fans, pumps, compressors that transform electrical energy into kinetic, potential and, via the losses in these processes, into thermal energy of fluids. The other group is heat exchangers. These energy transformers are usually integrated into the ventilation air handling unit (AHU). In modern AHUs so-called thermodynamic or active recovery systems [2], heat exchangers, and heat pumps are devices connected in terms of such energy transformation processes. In order to improve the efficiency of energy analysis of such various devices and the processes taking place therein, thermodynamic analysis is usually used along with the methods applied therein, such as exergy analysis, entropy generation minimization, and thermo-economics [3].

Thermodynamic analysis applied in scientific research that involves the First and Second Laws of Thermodynamics (FLT and SLT) allows to assess the performed processes in terms of quantity and quality. When applying the SLT, several values that define degradation of energy are encountered, for example, entropy and exergy [4,5], and, more rarely, entransy [6,7]. These indicators allow to demonstrate the true potential of a thermal system in terms of performance, therefore they become important when analyzing and comparing energy systems that use various types of energy [8]. However, applying each aforementioned value in practice leads to difficulties. Since entropy is the measure of disorder and unavailability of a system as well as an extensive state quantity, it can be sometimes difficult to interpret and understand, since it cannot be directly measured [9]. In addition to this, exergy analysis and its indicators are not extensively used in the building industry, designing energy efficiency of buildings or energy certification [10,11].

Exergy is understood as a part of energy that can be used for work relative to the reference state (or dead state) condition. It can also be defined as the maximum net useful work obtained from the available energy on the basis of the thermodynamic efficiency of Carnot cycle operating between the temperature of the systems and the reference environmental temperature [12]. Thus, exergy can be equated to available energy [13,14]. Therefore, it combines FLT and SLT through a reference environment [15]. In this case, it is necessary to assess the characteristics and change of the surrounding environment, because the obtained exergy results are the function of the selected reference environment [16,17,18]. Thus, when analyzing the processes of heat and work transformation in engineering systems of buildings, the issues surrounding the change of the reference conditions are relevant [19]. It should especially be taken into account when the analyzed processes take place at near environmental temperatures. Processes that take place in AHUs have these exact characteristics [20]. However, when performing exergy research, different authors interpret environmental conditions differently, sparking scientific discussion [15]. In some cases, reference parameters are variables [20,21,22], and in others they are constant, e.g., the average reference temperature and pressure [23]. It is also noted that the use of design or mean outdoor temperatures for exergy analysis may lead to significant uncertainties [21]. When assessing the energy system of a building in a selected timeframe in terms of exergy, e.g., ventilation AHU, it is noted that the maximum energy consumption occurs only at a specific reference temperature [22,24] and does not necessary correspond with the design temperature for energy calculations.

When linking entropy to the definition of exergy, it is known that entropy generation correlates with exergy destruction [6,25,26] as exergy destructions measure the real inefficiency of the system [27]. Exergy analysis also often assesses the entropy portion by including irreversibility [14]. The definitions of entropy and exergy and the links between them are provided in [6,12,14,28].

One of the main indicators used in exergy analysis that demonstrates system performance is exergy efficiency. It shows the irreversibility level that occurs during the process. Low exergy efficiency shows improper use of the energy sources [29] and could be used to select and design HVAC systems [11]. However, the use of this thermodynamic indicator still causes a lot of discussion and there exists several definitions of it [29,30,31].

Both exergy analysis and entropy generation analysis allow to determine the most efficient process [14,18]. The authors usually choose exergy analysis in order to compare and find the more efficient, better energy conversion process or system [10,32,33,34]. The thermodynamic or exergy analysis in terms of building systems is usually applied for systems analysis: Building envelope, HVAC systems [29,35] or components, e.g., heat exchangers [4,20,36,37], energy storage technologies, renewable and non-renewable energy sources [23,38], design optimization and control [39]. The carried out research of the IEA (International Energy Agency) lists recommendations and guidelines for designing low exergy buildings [17,40]. Research focused on such systems has been published in [21,41].

Meanwhile the amount of entropy in energy systems is often assessed by performing entropy generation minimization in order to assess and optimize the functioning of a system [25], to determine its designing procedure and sizing devices [42]. However, this optimization will not always lead to the optimal value of the design objective. In addition to this, it has been determined that “there is no direct monotonic relation between the minimum entropy generation rate and the best transfer performance of heat exchangers” [25]. Exergy analysis may not always be aimed at exergy destruction minimization, but instead be focused on the inlet and/or outlet exergy minimization [16], and the highest exergy efficiency of the system.

The abundance of research in this field shows the benefits of thermodynamics and a real need to evaluate the practical application of low-exergy systems [23]. Considering previous studies, it is obvious that the methods used for exergy analysis of energy transformation technologies, first and foremost—the methods used to determine exergy efficiency, should be improved in order to increase the consistency and universality of the methodology. The assumptions should be linked to the limitations of principles of thermodynamics and the inherent characteristics of exergy. In addition to this, proper attention should be paid to the evaluation of reference environment. Such methodology (calculation procedure, algorithms) improves the potential of applying exergy analysis in the practical research of building microclimate systems. Consistent analysis of processes taking place therein lays down the foundation for preparing the methodology of thermodynamic (exergy) analysis aimed at assessing momentary and seasonal efficiency of air handling units and other HVAC systems, taking into account varying reference environment temperatures [24,43].

This paper presents the thermodynamic analysis of an air handling unit (integrated heat recovery unit and heat pump) of a modern HVAC system. The Coefficient of Performance (COP), the produced entropy, destroyed exergy, and exergy efficiency are selected as the main indicators for the analysis. The transformation processes that take place in the separate components (heat exchangers) of the AHU at varying reference temperatures are analyzed in detail. The novelty of this study is demonstrated by analyzing the ventilation AHU using the developed methodology in a certain logical sequence. The presented logic of the solution can be integrated in dynamic modelling software by analyzing the transformative processes that take place not only in air handling units, but also in other HVAC systems.

## 2. Air Handling Unit as a Thermodynamic System

**Figure 1:**
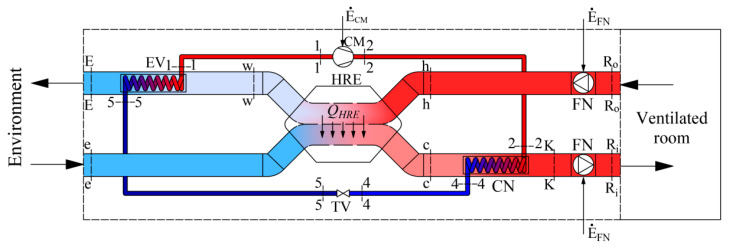
Ventilation AHU with an integrated HP and HRE.

The scheme of a modern air-handling unit (AHU) of a HVAC system is shown in Figure 1. Working fluids linked within energy transformation processes that take place in the AHU are the air that is necessary for ventilation and refrigerant/Freon. The latter device consists of a condenser (CN) and an evaporator (EV), which are connected by a compressor (CM) and a throttle valve (TV) to operate in the reverse cycle.

The AHU prepares the required amount of air ${\dot{M}}_{V}$ (for this paper a constant value was selected) of temperature *T_R_* required for ventilation, when the reference temperature is *T_e_*. In this case the AHU should provide fresh air required to ventilate the room with a specific heat flow:

(1) $${\dot{Q}}_{AHU}={\dot{M}}_{V}c_{p,a}(T_{R}-T_{e})$$

One of the peculiarities is that this heat flow changes corresponding with the reference temperature which depends on the local climate conditions. Therefore, the results of the thermodynamic analysis depend on the reference environment in a specific location, which the calculation method should reflect. This paper presents only the equations required for the exergy analysis of this AHU and concise commentary thereof.

AHU (Figure 1) consists of a heat recovery exchanger (HRE), heat pump (HP) and two fans (FN). The process of air preparation can be shortly described as follows. The amount of air required for ventilation ${\dot{M}}_{V}$ at temperature *T_R_* is supplied to and exhausted from the ventilated room. The outdoor air at temperature *T_R_* reaches the HRE, where it warms up from *T_e_* to *T_c_*. Then, in the HP condenser this air is warmed up to temperature *T_K_* and then slightly warms up in the supply fan (FN) and, having reached temperature *T_Ri_*, is supplied to the room. The air exhausted from the room at temperature *T_Ro_*, having warmed up to *T_h_*, in the HRE gives its heat to the supplied air and its temperature drops to *T_w_*. Next, in the HP evaporator the air continues to give away its heat by cooling to *T_E_* and is exhausted.

A process more linked to the HP could be defined as follows. The HP operates on the reverse cycle of Freon compression-expansion. This device warms up the outside air until it reaches the required temperature in the condenser (CN) and FN, having slightly warmed up the air, supplies it to the rooms. In the evaporator (EV) the cooling fluid evaporates due to the relatively high temperature of the exhaust air *T_w_*(see Figure 1).

At the level of the FLT, the energy transformation in heat exchangers is assessed using the heat transfer equation from the hotter working fluid *h* to the cooler fluid *c*:

(2) $${\dot{Q}}_{c}^{h}=AU\Delta T_{\ln m}$$

and the heat balance equation:

(3) $${\dot{Q}}_{c}^{h}={\dot{M}}_{c}c_{p,c}(T_{c1}-T_{c2})={\dot{M}}_{h}c_{p,h}(T_{h2}-T_{h1})$$

where *A* is the surface area, *U* is the overall heat transfer coefficient, Δ*T*_ln *m*_ is the logarithmic mean temperature difference, ${\dot{M}}_{c}$, ${\dot{M}}_{h}$ are the mass flow rates of cold and hot fluids, respectively, *c_p,c_*, *c_p,h_* are the specific heat capacities of cold and hot fluids, respectively, *T_c_*, *T_h_* are the temperatures of cold and hot fluids in the inlet and outlet, respectively.

**Figure 2:**
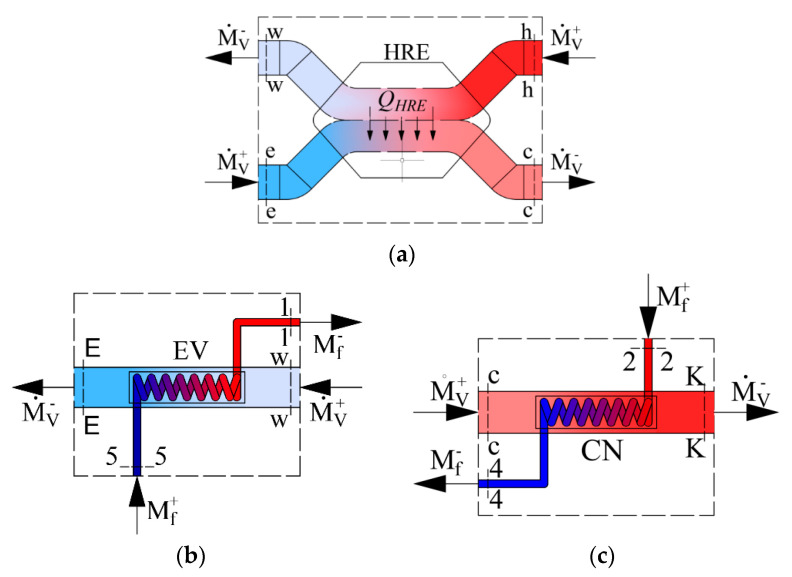
Schemes of energy calculations for components of the AHU: (**a**) Heat exchanger, (**b**) evaporator, (**c**) condenser. ${\dot{M}}_{f}$—the mass flow rate of Freon (kg/s); ${\dot{M}}_{V}$—the mass flow rate of the air used for ventilation (kg/s).

As the AHU heat exchangers we have a heat recovery exchanger, evaporator and condenser. Their schemes are shown in Figure 2 along with the corresponding energy balance equations, expressed in enthalpies:

- Heat recovery exchanger:
  (4a) $${\dot{M}}_{V}(h_{h}-h_{w})={\dot{M}}_{V}\left(h_{c}-h_{e}\right)$$
- Evaporator:
  (4b) $${\dot{M}}_{f}(h_{1}-h_{5})={\dot{M}}_{V}\left(h_{E}-h_{w}\right)$$
- Condenser:
  (4c) $${\dot{M}}_{f}(h_{2}-h_{4})={\dot{M}}_{V}\left(h_{K}-h_{c}\right)$$
  where *h* is enthalpy. Subscripts are used according to Figure 1 and Figure 2.

**Figure 3:**
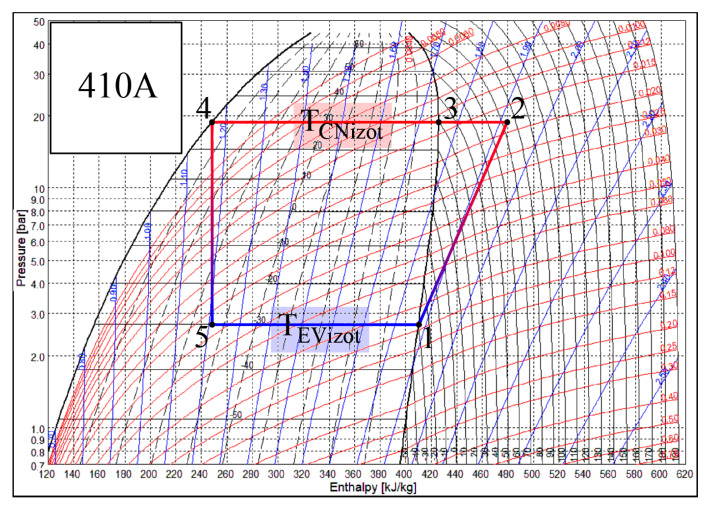
Temperature variation in the condenser (CN) and the evaporator (EV) of the heat pump. Specific numeric values correspond with the numeric case study analyzed below.

Heat transfer processes that take place in the CN and EV of the heat pump occur when states of air and Freon change. Each of these heat exchangers has its own specifics. There are different working fluids in the evaporator and the condenser, Freon and air. The change of the state of Freon is depicted in Figure 3. The SOLKANE Refrigerants [44] calculation program is used to represent the thermodynamic cycle of the heat pump. If Freon in the evaporator receives heat from the air (the temperature of the air is higher), then in the condenser the air receives heat from Freon (the temperature of the air is lower). For the most part of the process the state of Freon changes in accordance with the isotherm: *T_f_*_5_–*T_f_*_1_ in the evaporator and *T_f_*_3_–*T_f_*_4_ in the condenser.

The smaller part of the heat flow in the condenser is transferred as the state of the overheated vapor of Freon changes, i.e., as its temperature drops from *T_f_*_2_ to *T_f_*_3_. *T_e_*, along with the reference temperature, is always below the temperatures of the working fluids in the condenser. The problem of the varying reference temperature discussed below is not relevant to it. Meanwhile *T_e_* in the evaporator in the majority of engineering solutions for the AHU is between the temperatures of the Freon and air. As a result, in this case, the interpretation of directions of exergy flows should be given appropriate attention.

HRE is a specific heat exchanger. In it, heat exchange takes place between the air flows of the same mass flow rates: The removed air warms up the replacing, supplied air. In engineering practice, the efficiency of the HRE is then calculated using equations:

(5a) $$\varepsilon_{T}=\frac{T_{h1}-T_{h2}}{T_{h1}-T_{c1}}$$

or

(5b) $$\varepsilon_{T}=\frac{T_{c2}-T_{c1}}{T_{h1}-T_{c1}}$$

If, in AHU technological systems, the air supplied to the HRE is often at outdoor temperature *T_c_*_1_ = *T_e_*, then the indices in the equations change accordingly, corresponding with Figure 1:

(6a) $$\varepsilon_{T}=\frac{T_{h}-T_{w}}{T_{h}-T_{e}}$$

or

(6b) $$\varepsilon_{T}=\frac{T_{c}-T_{e}}{T_{h}-T_{e}}$$

The following AHU performance indicators were selected for this paper: Coefficient of performance (COP) and exergy efficiency *η_ex_*. AHU performance does not go beyond the FTL and, by essence, corresponds with the definition of the COP of a heat pump that considers the equivalence of electricity and exergy and is widely used in engineering practice:

(7) $$COP_{AHU}=\frac{{\dot{Q}}_{AHU}}{{\dot{E}}_{AHU}^{+}}$$

where ${\dot{E}}_{AHU}^{+}$ is the flow of exergy given outside of the boundaries of the AHU as a thermodynamic system both in the form of work and heat. It can be used by the fans, compressor, and additional heaters or coolers not shown in the scheme that use external working fluids or electricity. This COP is slightly different from the COP of AHUs that use only electricity as their denominators, which have been becoming more and more popular in the engineering practice. While it does constitute the net exergy flow, other possible energy inputs (cooling, heat) should be considered on the basis of their thermodynamic value (exergy).

Exergy efficiency, discussed below, is another AHU performance indicator. In the most general case (for the entire AHU or its components), it would be the ratio of exergy that leaves (results from) the analyzing system ${\dot{E}}_{{}_{}}^{-}$ to the exergy that this system is supplied with, ${\dot{E}}_{{}_{}}^{+}$:

(8) $$\eta_{ex}=\frac{{\dot{E}}_{{}_{}}^{-}}{{\dot{E}}_{{}_{}}^{+}}\;\mathrm{or}\;\eta_{ex}=1-\frac{{\dot{L}}_{c}^{h}}{{\dot{E}}_{{}_{}}^{+}}$$

where ${\dot{L}}_{c}^{h}$ is exergy destroyed in the process.

Exergy is a thermodynamic property, dependent on the state of the analyzed system and its surrounding environment, the so-called reference environment or, for thermal systems—reference temperature (RT). Determining exergy efficiency becomes complicated when RT varies and could be below, above or equal to the operating temperature of the working fluids, which is exactly the case for the AHU.

## 3. The Specifics of Exergy Analysis in HVAC Processes

In sections below we will discuss several specific questions: The variation of the direction of exergy flows of a stationary heat transfer process and the variation of the exergy efficiency of such process that changes the RT. Two approaches to determining the amount of produced energy in a heat exchanger that transfers heat in a stationary manner are compared below.

### 3.1. Produced Entropy Calculation Approaches for Heat Exchangers

**Figure 4:**
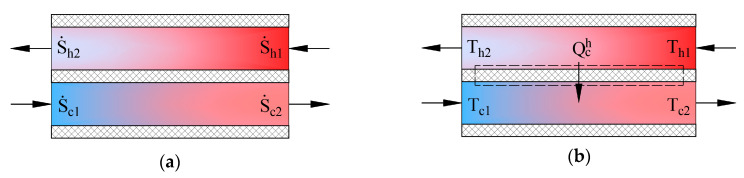
State parameters for determining produced entropy in a counter-current heat exchanger. (**a**) entropies of working fluids; (**b**) temperatures of working fluids.

Heat exchangers in AHUs are important energy transformers. Figure 4 shows schemes of counter-current heat exchangers where the process of heat transfer is defined in two ways. The picture on the left (a) shows entropies of flows $\dot{S}={\dot{M}}_{i}s_{i}$, in this case the produced entropy is determined by using:

(9) $$\Delta {\dot{S}}_{c,irr}^{h}={\dot{S}}_{h1}+{\dot{S}}_{c1}-{\dot{S}}_{h2}-{\dot{S}}_{c2}$$

In the second case the process (b) is defined by the transferred heat flow and the input and output temperatures of working fluids. The expression of entropy in the heat transfer process is $dS^{q}=\delta Q/T$. The produced entropy is expressed as the difference between the average entropy of the cooler working fluid and average entropy of the hotter working fluid:

(10) $$\Delta {\dot{S}}_{c,irr}^{h}=\frac{1}{2}\left(\frac{{\dot{Q}}_{c}^{h}}{T_{c1}}+\frac{{\dot{Q}}_{c}^{h}}{T_{c2}}\right)-\frac{1}{2}\left(\frac{{\dot{Q}}_{c}^{h}}{T_{h1}}+\frac{{\dot{Q}}_{c}^{h}}{T_{h2}}\right)$$

In the condenser, this value would be determined by adding the values of the cooling of overheated Freon vapor and Freon condensation. The results can be linked to the exergy analysis to find the destroyed exergy, which is generally expressed as follows:

(11) $${\dot{L}}_{c}^{h}=T_{e}\Delta {\dot{S}}_{irr}\geq 0$$

Having combined these equations, the destroyed exergy in the process in case (b) can be expressed using the Carnot factor values $\eta_{Ci}=1-\frac{T_{e}}{T_{i}}$ in the following manner:

(12) $${\dot{L}}_{c}^{h}={\dot{E}}_{h}^{}{}_{}-{\dot{E}}_{c}^{}{}_{}=\int_{h2}^{h1}\left(1-\frac{T_{e}}{T_{h}}\right)\delta {\dot{Q}}_{c}^{h}-\int_{c1}^{c2}\left(1-\frac{T_{e}}{T_{c}}\right)\delta {\dot{Q}}_{c}^{h}$$

**Figure 5:**
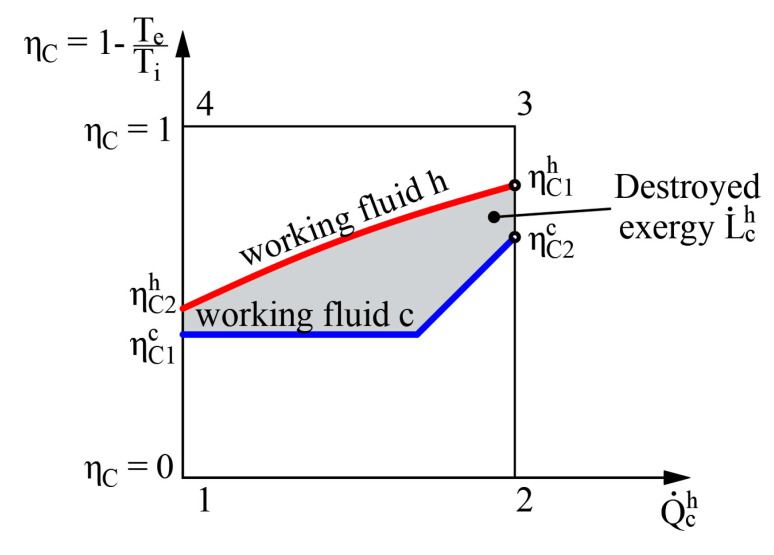
Graphical representation of destroyed exergy ${\dot{L}}_{c}^{h}=f(\eta_{Ci},{\dot{Q}}_{c}^{h})$ in the heat transfer process in the heat exchanger.

Graphical interpretation of this expression is shown in Figure 5.

The left side of Equation (12) shows the amount of exergy flow given to the system while the right side shows the obtained amount of exergy flow. It should be noted that regardless of where the value $\eta_{C}$ = 0 is in terms of other $\eta_{Ci}$ (e.g., between the values of hot and cold working fluids), the destroyed exergy that defines this process is still the area between these two curves (see Figure 5).

The method to determine the destroyed exergy depends on the available state parameters. In the first case problems might arise due to the difference of reference states of entropies for various working fluids. On the basis of the obtained indicator for destroyed energy it is convenient to compare devices of similar efficiency. However, there is no universal performance indicator available in this case. In order to determine exergy efficiency *η_ex_*, along with $\Delta {\dot{S}}_{irr}$ or ${\dot{L}}_{c}^{h}$ at least the exergy flow that is supplied to the system should be known.

The case for AHU is special because the varying RT has a significant impact on the procedures of exergy analysis. It is obvious that when RT changes, produced entropy $\Delta {\dot{S}}_{irr}$ does not change; therefore, the results of the analysis that only indicate produced entropy become limited. As demonstrated below, even when the rate and direction of the heat flow is stable, when the RT changes, the exergy flow also changes in terms of its rate and direction. 

### 3.2. Exergy Efficiency of Stationary Heat Transfer Process at Varying RT

**Figure 6:**
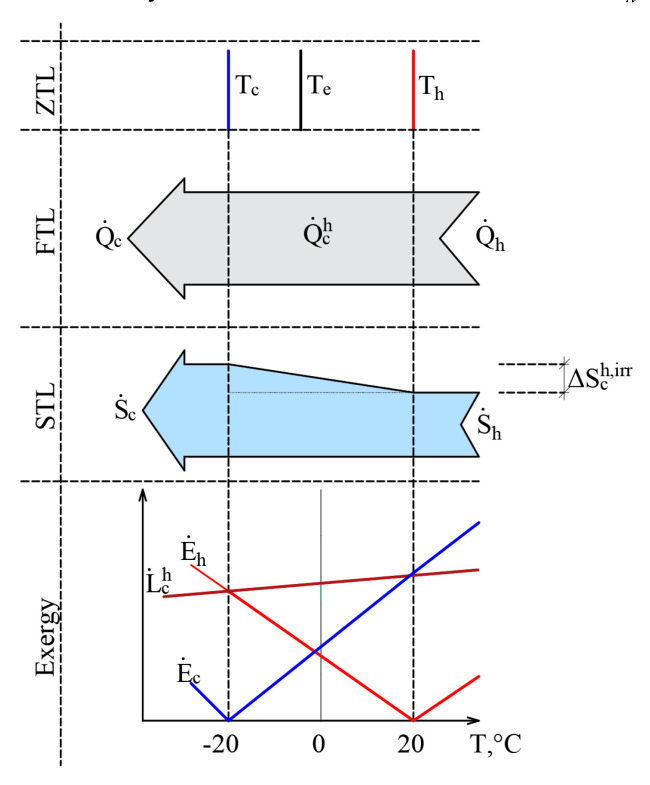
Heat transfer process from the positions of the ZLT, FLT, SLT and exergy when reference temperatures are close to the temperatures of heat sources.

The authors have published papers on the impact of the varying RT on the direction of the exergy flow when the heat flow is stable [20,45]. Here, we have a stationary heat transfer process that is caused by stable temperatures *T_h_* = 20 °C and *T_c_* = –20 °C. The selected temperatures are close to the natural environment. The process is shown in Figure 6 from the point of view of the Zero Law of Thermodynamics (ZLT), the FLT, the SLT, and exergy.

It should be noted that the heat transfer process itself is not related to outdoor air temperature, i.e., *T_c_* ≠ *T_e_* or *T_h_* ≠ *T_e_*. In addition to this, in the cases of the ZTL, the FTL, and the STL *T_e_* is not a parameter that affects the process indicators. In terms of the FTL there is a stable heat flow ${\dot{Q}}_{c}^{h}=const$, while in terms of the STL, the produced entropy is $\Delta S_{c}^{h}{}_{irr}=const.$ The RT, i.e., outdoor air temperature *T_e_*, here on the x-axis shown as a variable and is prominent in the exergy analysis of the process. The exergy balance equation of the process discussed herein is ${\dot{E}}_{h}^{}{}_{}={\dot{E}}_{c}^{}{}_{}+{\dot{L}}_{c}^{h}$. The dependence of these three members on *T_e_*, is shown in the portion of the figure that is dedicated to exergy. Based on Equation (11), the destroyed exergy is linearly linked to $\Delta S_{c}^{h}{}_{irr}$ and in all cases, ${\dot{L}}_{c}^{h}\geq 0$. Within the temperature range $T_{h}\geq T_{e}\geq T_{c}$ exergy flows formed by both temperatures are given to the system ${\dot{E}}_{h}^{+}$ and ${\dot{E}}_{c}^{+}$, which are denoted with a superscript index “+”. There is no resulting exergy flow that leaves the system here. We have a case of ${\dot{E}}_{h}^{+}+{\dot{E}}_{c}^{+}{{}_{}}_{}={\dot{L}}_{c}^{h}$.

This interpretation shows how in a stationary heat transfer process, formed by temperatures *T_h_* and *T_c_*, the direction of exergy flows depends on the position of *T_e_* with respect to these temperatures. In other words, both exergy flows that characterize the heat transfer process are always directed at RT and follow it as it changes.

**Figure 7:**
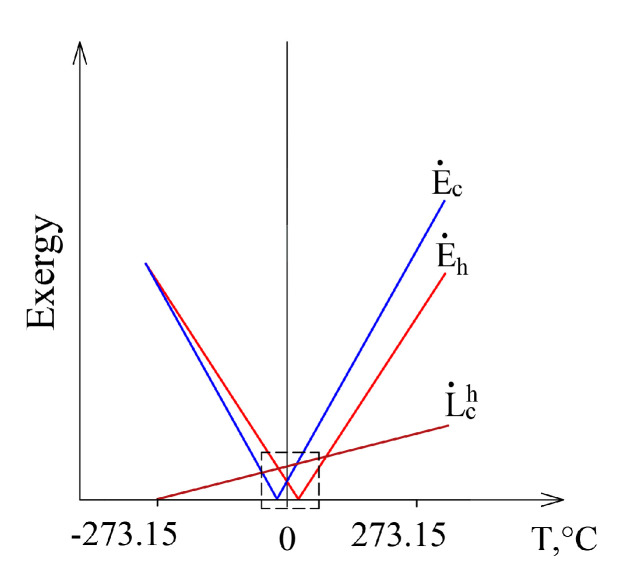
The members of the exergy balance of the heat transfer process in a wide range of *T_e_.*

This case, having expanded it to conditions that are marginal in terms of thermodynamics from *T_e_*= 0 K to *T_e_* >> *T_h_*, is shown in Figure 7. Exergy flow data, shown in Figure 6, is denoted by a dashed line. When *T_e_* equals any of these temperatures (*T_e_ = T_h_* or *T_e_ = T_c_*), then the corresponding exergy equals 0. Destroyed exergy is proportional to *T_e_* and always above 0.

**Figure 8:**
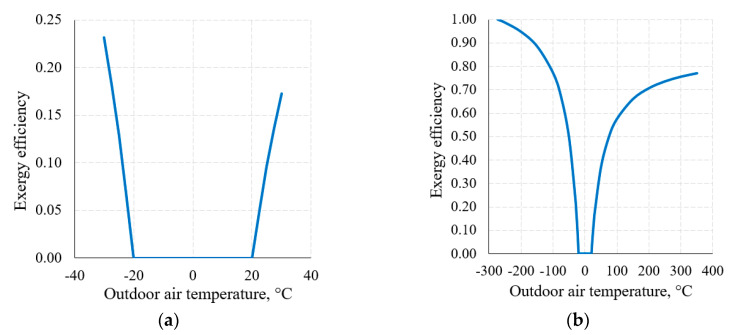
Exergy efficiency of the heat transfer process between temperatures *T_h_* = 20 °C, *T_c_* = −20 °C at a varying RT: (**a**) 30 °C ≥ *T_e_* ≥ −30 °C; (**b**) from *T_e_* = 0 K to higher values.

An important attribute of the thermodynamic process in exergy analysis is the thermodynamic (exergy) efficiency of that process (Equation (8)). The nature of the change of this indicator for the heat transfer process (when *T_h_* = 20 °C and *T_c_* = −20 °C) in the thermodynamic system shown in Figure 6, within the reference temperature range 30 °C ≥ *T_e_* ≥ −30 °C, is shown in Figure 8a.

Figure 8b shows this indicator when the RT range, on the basis of the thermodynamic interpretation, is expanded up to the theoretically possible range. Here we can see that the dependency has the maximum *η_ex_* = 1 and minimum *η_ex_* = 0 values; in addition to this, it is generally not symmetrical with respect to 0 °C (the average value of *T_h_* = 20 °C and *T_c_* = −20 °C). The maximum value always corresponds with *T_e_* = 0 K. The minimum value *η_ex_* = 0 is always within the range between *T_e_* = *T_h_* and *T_e_* = *T_c_*, which, in this case, is (*T_h_* = 20 °C, *T_c_* = −20 °C).

Case ${\dot{E}}_{h}^{+}={\dot{E}}_{c}^{+}{{}_{}}_{}$ (the intersection of exergy flows in Figure 6), quantitatively, is not symmetrical in terms of temperatures (i.e., does not equal 0 °C in this case) and is the so-called harmonic mean $T_{e,E_{h}=E_{c}}=\frac{2T_{h}T_{c}}{T_{h}+T_{c}}$, different for each combination of these temperatures. In the numeric case analyzed herein it equals −1.46 °C.

The presented numeric case of the heat transfer process shows that such interpretation of the direction of an exergy flow corresponds with the fundamental axioms of thermodynamic (exergy) analysis. First of all, exergy losses are always ${\dot{L}}_{}\geq 0$ and when $\Delta {\dot{S}}^{i}=const$ they are proportional to *T_e_*. In addition to this, the exergy efficiency of a system and a process taking place therein is $0\leq \eta_{ex}\leq 1$. Therefore, the thermal exergy flow moves from the thermal source to the environment. The solution of an element developed hereby can be reliably used in the thermodynamic (exergy) analysis of the energy transformation chain of HVAC systems.

### 3.3. Features of Exergy Analysis of Heat Exchangers in the AHU

**Figure 9:**
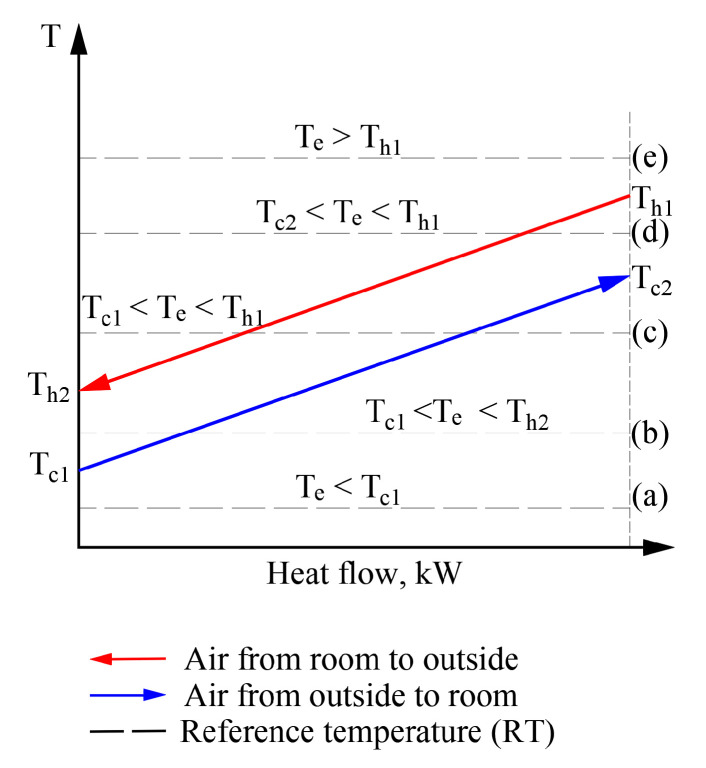
Possible positions of temperatures of working fluids and outdoor air with respect to each other in heat exchangers of the AHU.

The cases discussed above occur in HVAC systems and determining exergy efficiency becomes problematic when RT varies and could be below, above, or equal to the operating temperature of the working fluids. Figure 9 shows a sequence of RT positions for a heat exchanger that operates in a stationary mode (i.e., different temperatures of working fluids do not change their values; instead, RT positions change with respect to them).

In a real exergy analysis task of HVAC, the variation of the RT has its own behavior over time (which depends on the momentary climate conditions) which can affect the temperatures of working fluids as well. The usual sequence of exergy assessment and calculation, even at a varying RT, is acceptable without the specific properties for cases (a) and (e). In these cases, the numeric value of RT equals, is above or below the temperatures of any working fluid. However, in order to move to a thorough analysis of processes at a varying *T_e_*, an algorithm is required to solve the separate cases shown here (e.g., (b), (c), and (d)).

Furthermore, we analyze the HRE, i.e., an air-to-air heat exchanger where ${\dot{M}}_{h}={\dot{M}}_{c}={\dot{M}}_{V}$. Process flows in a HRE that is typical of an AHU do not directly cross the boundaries of the thermodynamic system shown in Figure 1; however, the HRE is a very important component to the general performance of the AHU. When performing the analysis of AHU components, a methodology for the thermodynamic analysis of the HRE at a varying RT when its values cross the temperatures of working fluids was prepared. Paper [45] proposes a solution using the Carnot factor while in Reference [20,46] coenthalpies were applied. In addition to this, the methodology for the HRE for determining universal and several functional exergy efficiencies was developed in Reference [46]. In order to avoid repeated publication of the same material, the authors only reference the papers that present the aforementioned methods. Only a succinct definition of coenthalpy is presented. According to Borel [47,48], in exergy analysis that combines the FLT and the SLT, coenthalpy is the potential of exergy flow (and, at the same time, the state parameter). The coenthalpy of state *i* of the working fluid (its mass flow) (derivative status parameter from enthalpy $h_{i}=c_{p}T_{i}$ and entropy $s_{i}=c_{p}ln\frac{T_{i}}{273.15}$) at reference temperature *T_e_* is calculated using equation:

(13) $$k_{i}=h_{i}-T_{e}s_{i}=c_{p}(T_{i}-T_{e}ln\frac{T_{i}}{273.15})$$

where *h_i_* is the enthalpy of state *i* of the working fluid, *c_p_* is the specific heat capacity.

Reference coenthalpy *k_e_* that corresponds with the reference temperature *T_e_* is determined as follows:

(14) $$k_{e}=T_{e}c_{p}(1-ln\frac{T_{e}}{273.15})$$

Then, the compared exergy flows *i*, conventionally used (calculated and analyzed) in exergy analysis, are:

(15) $$e_{i}=k_{i}-k_{e}=\left(h_{i}-h_{e}\right)-T_{e}(s_{i}-s_{e})$$

As the process shifts from state “1” to state “2”, it is defined by the difference between potentials (coenthalpies) of the exergy flow:

(16) $$e_{12}=k_{1}-k_{2}=\left(h_{1}-h_{2}\right)-T_{e}\left(s_{1}-s_{2}\right)\;\mathrm{or}\;{\dot{E}}_{12}={\dot{M}}_{12}\left(k_{1}-k_{2}\right)={\dot{K}}_{1}-{\dot{K}}_{2}$$

Accordingly, for energy balance equations in Figure 2, exergy equations on the basis of coenthalpies for heat exchangers in the AHU are expressed as follows:

- HRE:
  (17a) $${\dot{M}}_{V}(k_{h}-k_{w})={\dot{M}}_{V}\left(k_{c}-k_{e}\right)+{\dot{L}}_{HRE}$$
- Evaporator:
  (17b) $${\dot{M}}_{f}(k_{1}-k_{5})={\dot{M}}_{V}\left(k_{E}-k_{w}\right)+{\dot{L}}_{EV}$$
- Condenser:
  (17c) $${\dot{M}}_{f}(k_{2}-k_{4})={\dot{M}}_{V}\left(k_{K}-k_{c}\right)+{\dot{L}}_{CN}$$

An analogous balance equation for the entire AHU:

(18) $${\dot{M}}_{V}k_{e}+{\dot{M}}_{V}k_{Ro}+{\dot{E}}_{CM}+2{\dot{E}}_{FN}={\dot{M}}_{V}k_{Ri}+{\dot{M}}_{V}k_{E}+{\dot{L}}_{AHU}$$

where ${\dot{M}}_{V}$, ${\dot{M}}_{f}$ are the mass flow rates of ventilated air and Freon, $\dot{L}$ is the destroyed exergy, $\dot{E}$ is the exergy flow rate, subscripts are used according to Figure 1.

On the basis of the numeric results of the case study below it will be demonstrated that appropriate approach to the directions of heat exergy flows and the determination of their values by combining the methodology based on the Carnot factor and the basis of coenthalpies allows to develop algorithms of dynamic modelling for HVAC systems, as well as to reflect the peculiarities of energy transformation processes in HVAC devices in a manner that is valid in terms of thermodynamics. 

## 4. Case Study—Results and Discussion

The stable air flow rate of the analyzed AHU is 560 m^3^/h and power input for fans (supply and exhaust) is 2 × 77 W. The efficiency of the HRE is 70%, the isentropic efficiency of the HP compressor is 0.8, and the power consumption efficiency of the fan is 0.82. The environment (as well as reference) temperature range is *T_e_* = −30, …, +10 °C while the room air temperature is 22 °C. Freon is 410a, isotherm in the evaporator is $T_{EVizot}=T_{f5}=T_{f1}$= −30 °C, isotherm in the condenser is $T_{CNizot}=T_{f3}=T_{f4}$= 30 °C (Figure 3.).

**Figure 10:**
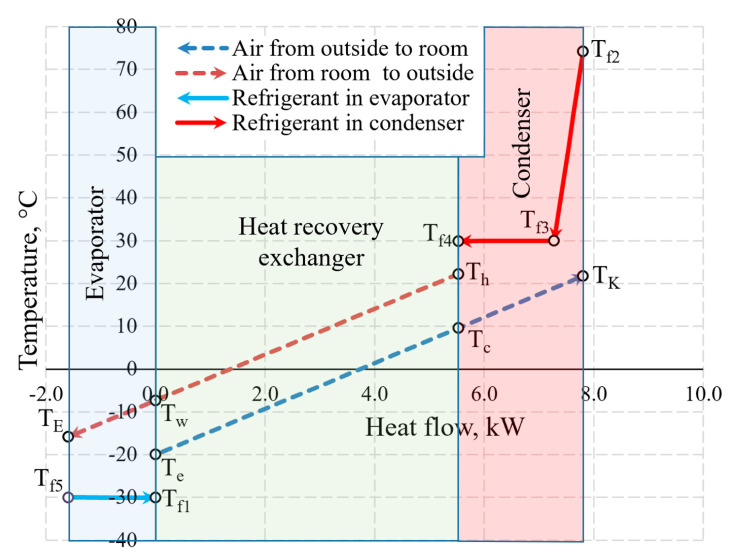
The variation of air and Freon temperature in the heat exchangers of the AHU when *T_e_* = −20 °C.

An integral chart of the variation of working fluids (air and Freon) of the AHU in the heat exchangers is shown in Figure 10. The depicted temperatures are within the range that is typical of processes of real-world ventilation and HP used for such ventilation. To the right of 0.0 on the *x*-axis the thermal input of the AHU is shown which is created by the HRE and the CN. To the left of 0.0 the thermal input of the evaporator that it receives from the exhausted air is shown.

The selection of the size of the AHU and *T_e_* = −20 °C does not have a numeric significance in terms of the discussed issues of exergy analysis from the methodical point of view. The warming up of air in fans has been assessed in the calculations but is not reflected in the chart directly due to relatively low numeric value.

**Figure 11:**
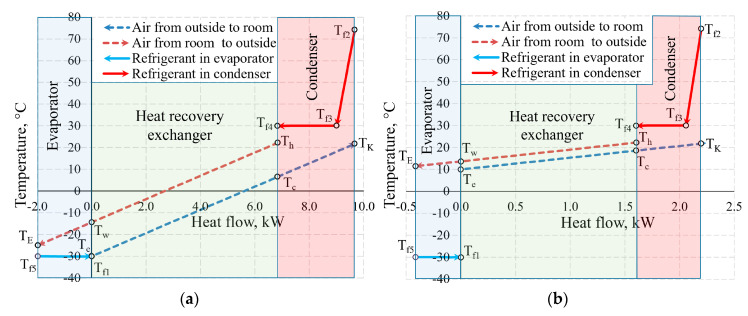
Temperatures in the heat exchangers and heat flows therein at outdoor air temperatures of (**a**) −30 °C; (**b**) +10 °C.

Figure 11 shows the temperature values of the same AHU in the heat exchangers at outdoor air temperature *T_e_* = −30 °C and 10 °C. Not only the positions of temperatures of the working fluid with respect to each other change, but also the heat flow transferred within them, which is shown on the x-axis at different scales. 

**Figure 12:**
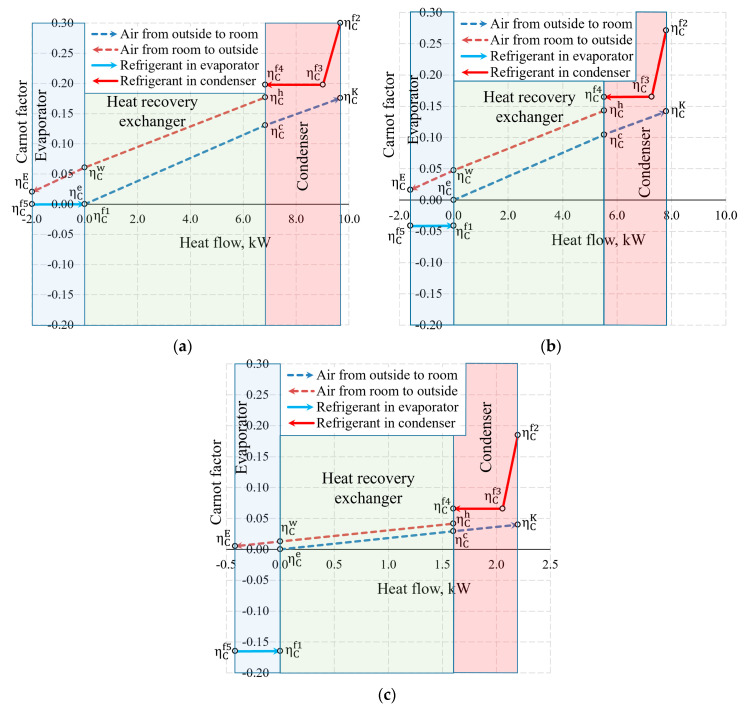
Carnot factors in heat exchangers and heat flows therein at outdoor air temperatures of (**a**) −30 °C, (**b**) −20 °C and (**c**) +10 °C.

The aforementioned Carnot and coenthalpy methods [45,46] were not used together for the same numeric case which will be performed herein. The cases of variations of the Carnot factor in these three temperature cases (*T_e_* = −30 °C, −20 °C and 10 °C) are shown in Figure 12.

It is observed that the Carnot factor is 0 in all cases for the outdoor air that enters the HRE. It equals 0 for the evaporator as when $T_{EVizot}=T_{e}$= −30 °C. The area between the Carnot lines within the input of the heat exchanger or any other general inputs shows destroyed exergy, as demonstrated in Figure 5. The case for +10 °C, with its relatively large area, draws attention and poses an objective—the reduction of exergy losses in the evaporator when the outdoor air temperatures are higher.

**Figure 13:**
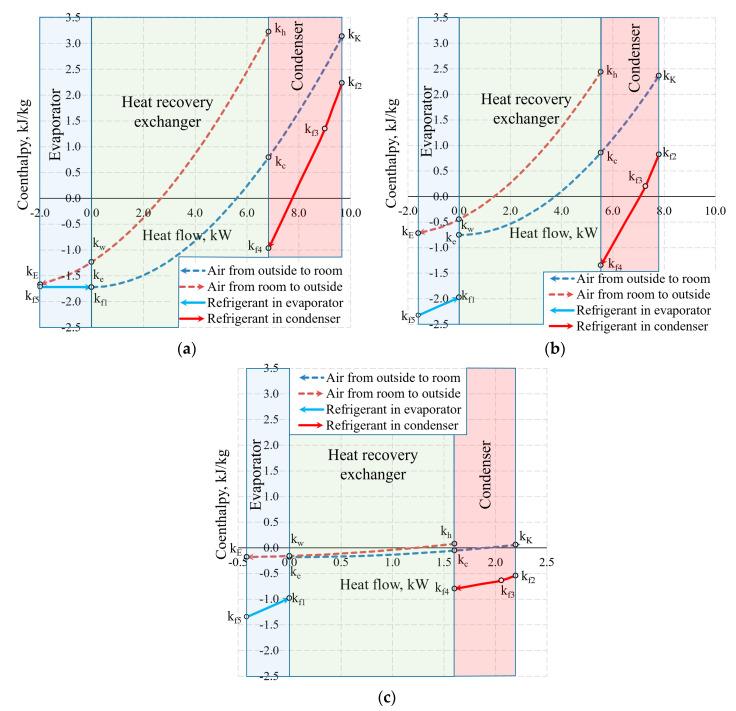
Coenthalpies and heat flows in heat exchangers at outdoor air temperatures of (**a**) −30 °C; (**b**) −20 °C, and (**c**) +10 °C.

Figure 13 shows the same temperature cases by expressing the states of heat exchangers in coenthalpies. It should be noted that in the exergy analysis of the HRE [20,46], the hot and cool working fluids of the heat exchanger are the air with the same mass flow rate, thus the results of the analysis are interpreted directly on the basis of the variation of the coenthalpies of the working fluid. When the HRE, EV, and CN are assessed in an integral manner, this advantage disappears.

Based on our selection, we have an air handling unit with a stable mass flow rate ${\dot{M}}_{V}$, however, the mass flow rate of the HP (i.e., the same for the evaporator and the condenser) depends on *T_e_*. In addition to this, the reference state parameters for the thermodynamic state parameters (enthalpy and entropy) of these different working fluids are autonomous. In this stage, air coenthalpies have been chosen for graphical representation while the Freon coenthalpies have been reduced at a ratio of ${\dot{M}}_{f}/{\dot{M}}_{V}$.

The exergy balance equation for the condenser (Equation (17c)) in this case would be $\left({\dot{M}}_{f}/{\dot{M}}_{V}\right)k_{2}-\left({\dot{M}}_{f}/{\dot{M}}_{V}\right)k_{4}=\left(k_{K}-k_{c}\right)+l_{CN}$.

Coenthalpies of Freon reduced in this manner allow to compare the values of exergy flows of the HRE, EV and CN diagrammatically. It should be noted that the area *k* = *f*(*Q*) between coenthalpies does not have a clear physical, thermodynamic equivalent, unlike the equivalent for the destroyed exergy in the case of Carnot factor. The fundamental quantitative indicator is the difference between the coenthalpies of a specific heat exchanger that corresponds with the released or received exergy flow. It should be mentioned that the value of coenthalpy that decreases in the direction of the flow of the working fluid in the process (e.g., a specific heat exchanger) shows that the system is supplied with an exergy flow. If the coenthalpy increases in the direction of the flow of the working fluid, it means that the system supplies the heat exchanger with an exergy flow. A typical case in an evaporator at −30 °C is when the coenthalpy does not change due to $T_{EVizot}=T_{e}$ remaining for the entirety of the process.

**Figure 14:**
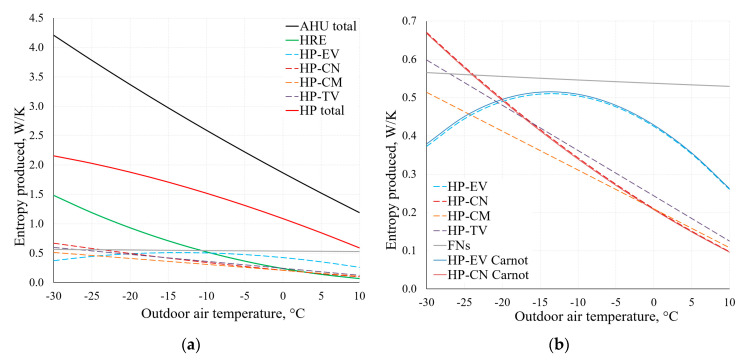
Entropy produced in AHU components $\Delta {\dot{S}}_{irr}$ at different reference temperatures (**a**). Chart (**b**) shows an extract from the lower part of chart (**a**), using Equations (9) and (10).

By using the applied analysis of the AHU, Figure 14 depicts the change of the produced entropy in the range of *T_e_* = −30 °C, …, +10 °C. These results have been obtained by using Equations (9) and (10).

As observed in the comparison of the results of condenser (CN) and evaporator (EV) within the range of temperatures typical of HVAC depicted in Figure 14b, there is no significant difference between these results. The results are analogous for other components of the AHU, which shows that when using dynamic modelling algorithms, the method that is more suitable in terms of the available data can be chosen.

**Figure 15:**
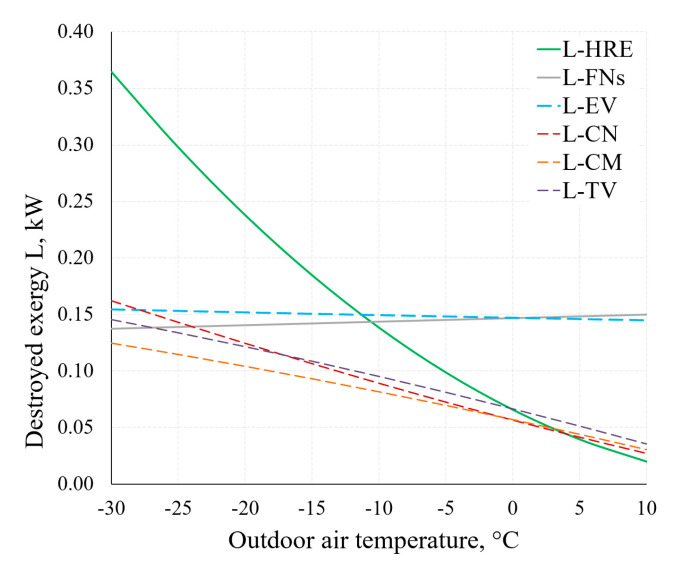
Destroyed exergy $\dot{L}$ in the AHU components HRE, EV, CN and FNs at different RT.

Figure 15 shows the destroyed exergy in several components of the AHU. Considering the operation of the AHU under real-world conditions (in a wide range of outdoor air temperatures), the decreasing values of destroyed exergy of the HRE and CN, yet not decreasing values of destroyed exergy of the EV and FNs as the outdoor air temperature increases should be noted. Therein lies the potential of improving the general indicators of the thermodynamic efficiency of AHUs.

**Figure 16:**
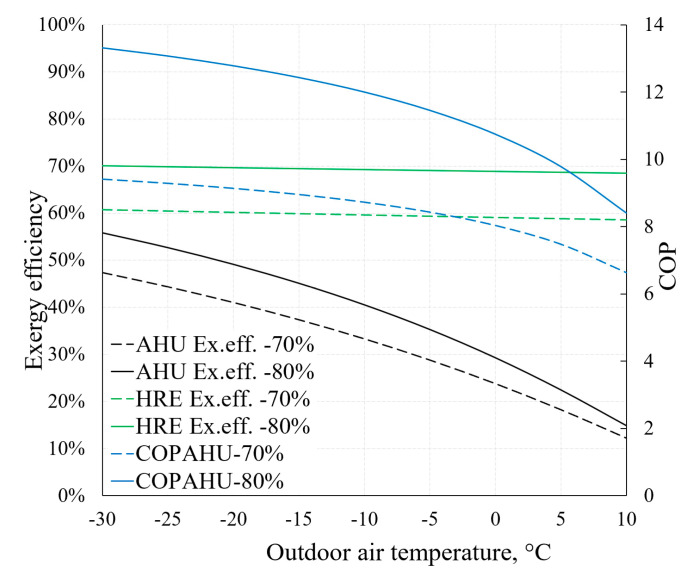
Dependence of COP and exergy efficiency of AHU on reference temperature.

On the basis of the previously described methodical foundation we can obtain AHU indicators relevant to HVAC design that are the focus of dynamic modelling, such as *COP_AHU_* (Equation (7)) or exergy efficiency *η_ex_* (Equation (8)). The dependence of these indicators on outdoor air temperature (RT) is depicted in Figure 16. It also illustrates the aforementioned statement that the effectiveness of the HRE (Equations (5) and (6)) has an important role regarding AHU performance indicators. For this purpose, two cases are presented: When *ε_T_* equals 70% and 80%. 

Higher *ε_T_* allows to achieve better AHU performance indicators, especially at lower reference temperature values. It can be noted that the exergy efficiency of the HRE remains nearly stable (in both cases of *ε_T_*) due to the fact that it is supplied with the air at reference temperature. The exergy efficiency of the entire device responds more to the reference temperature by dropping several times in the analyzed range (from –30 °C to +10 °C). It occurs mostly due to destroyed exergy in the evaporator and fan being almost independent of the reference temperature (see Figure 15). Additional calculations show that without the HRE, the performance coefficient of AHU would practically correspond with the HP and in this numeric case would be almost 3.

We hereby list the aforementioned specifics of processes in HVAC devices. First of all, the parameters of working fluids change and depend on the constantly changing outdoor air temperature *T_e_*, which, in terms of exergy analysis, is reference temperature. In a specific location the variation of temperature is typical of that location only. The assessment and choice of solutions in terms of engineering and economy depends on a longer period, seasonal processes and indicators of device performance that can be only revealed by employing dynamic process modelling. One of its main components is exergy analysis. The peculiarities of processes in AHUs are listed and demonstrated therein. The reflection of these specifics in dynamic modelling algorithms enhances the application of exergy analysis in the assessment and choice of HVAC systems.

## 5. Conclusions

HVAC systems use about one third of global final energy while energy transformation processes therein often take place all-year-round. These are the processes of heat transfer and power use for circulation of working fluids. As a general rule, the quantitative and qualitative parameters of these processes change depending on the constantly changing reference temperature. Such scope and constant operation raises the goal of energy efficiency for HVAC systems and the challenge of using thermodynamic (exergy) analysis to assess such efficiency. The fact that the reference temperature for exergy analysis in the same device can simultaneously be below, above or equal to the different operating temperature of the working fluids raises an additional challenge. This requires a thorough and universal approach to the change of the direction of thermal exergy flows and poses the objective of preparing algorithms that represent such approach unambiguously.

The performed case study of an air handling unit has shown that all these issues were discussed, expressed in an analytical manner and a logical sequence for performing the exergy analysis of this device was proposed and realized. It should be developed and applied to other HVAC systems.

The main methodical conclusions of the paper are the following:

1. Two exergy flows that characterize the transferred heat flow formed by two temperatures are always directed at the reference temperature and correspond with its change. Such interpretation of the direction of exergy as the reference temperature changes corresponds with the fundamental axioms of thermodynamic (exergy) analysis:
  (a) Exergy losses ${\dot{L}}_{}\geq 0$ when $\Delta {\dot{S}}_{irr}^{}=const$ are always proportional to *T_e_*;
  (b) the exergy efficiency of a system and the process taking place therein is $0\leq \eta_{ex}\leq 1$.
2. Even though there are two ways to determine the entropy produced during the heat transfer process (on the basis of entropy balance and Carnot factor), in order to determine the exergy efficiency of this process, at least one of the two exergy flows has to be known.
3. As the temperature change in heat exchangers is nearly rectilinear, i.e., when temperature changes are not significant, both the Carnot factor and methods based on coenthalpies can be used for exergy analysis. The decision depends on the information available.

The main conclusions with regards to the numeric case are the following:

1. Process parameters in the AHU and its HP components, as well as performance indicators thereof, change within the range of change of RT. There is a trend that as the RT increases, most of the indicators drop. However, to each component there a specific nature of variation of the produced entropy, destroyed exergy and exergy efficiency.
2. With regards to RT, heat pump evaporator and fans are distinguished by a constantly non-decreasing destroyed exergy indicator. Here exergy analysis shows the potential of improving general thermodynamic efficiency indicators of AHUs.
3. Even without being an indicator for AHU performance comparison, entropy generation shows the distribution of process irreversibility in components as well as the specifics of changes in processes. It also allows to verify the intermediate results of exergy analysis.
4. Within the selected, rather wide range of RT, the change in the AHU coefficient of performance remains quite high and drops 30% to 40% when RTs are higher. Absolute values are highly dependent on the effectiveness of the heat recovery exchanger.
5. Exergy efficiency of AHU in this range of RT of −30 °C, …, +10 °C drops from 45–55% to 12–15% even though the exergy efficiency of the HRE basically does not change. The main reason for this is the aforementioned stable (essentially independent of the RT) value of the destroyed exergy for evaporator and fans.

The issues analyzed in the paper, the demonstrated specifics of processes in AHU and the solution thereof in the case study show that using these methods for dynamic modelling algorithms enhances the application of exergy analysis in the assessment of HVAC systems and the choice of such systems.

## Nomenclature

Abbreviations	
AHU	Air Handling Unit
CM	Compressor
CN	Condenser
COP	Coefficient of Performance
EV	Evaporator
FLT	First Law of Thermodynamics
FN	Fan
HP	Heat Pump
HRE	Heat Recovery Exchanger
HVAC	Heating, Ventilation and Air Conditioning
RT	Reference Temperature
SLT	Second Law of Thermodynamics
TV	Throttle Valve
ZLT	Zero Law of Thermodynamics
Variables	
*A*	surface area (m^2^)
*c_p_*	specific heat capacity (kJ/kg·K)
*e*	specific exergy (kJ/kg)
$\dot{E}$	exergy flow rate (kW)
*h*	enthalpy (kJ/kg)
$\dot{K}$	coenthalpy rate (kW)
*k*	specific coenthalpy (kJ/kg)
*l*	specific destroyed exergy (kJ/kg)
$\dot{L}$	destroyed exergy (kW)
$\dot{M}$	mass flow rate (kg/s)
$\dot{Q}$	heat transfer flow rate (kW)
*R_i_*	inlet to room (K)
*R_o_*	outlet from room (K)
$\dot{s}$	specific entropy (kJ/kg·K)
$\dot{S}$	entropy (kW/K)
*T*	temperature (K)
*U*	overall heat transfer coefficient (W/m·K)
Δ	difference (–)
$\varepsilon_{T}$	effectiveness of heat recovery exchanger (–)
$\eta_{C}$	Carnot factor (–)
*η_ex_*	exergy efficiency (–)
Subscripts	
*a*	air
*AHU*	air handling unit
*c*	cooler fluid
*e*	state of reference environment
*CNizot*	isotherm in the condenser
*EVizot*	isotherm in the evaporator
*f*	Freon / refrigerant
*h*	hotter fluid
*i*	state of fluid
*irr*	produced / irreversible
ln *m*	logarithmic mean
*R*	room
*V*	ventilation
*w*	warm exhausted air after HRE
1	in
2	out
Superscripts	
*h*	hotter fluid
*q*	heat transfer
+	to the system
–	out of the system
